# Unveiling the Effects of Interval Resistance Training and Chlorella Vulgaris Supplementation on Meteorin-like Protein and Oxidative Stress in Obese Men

**DOI:** 10.1016/j.cdnut.2024.104428

**Published:** 2024-07-25

**Authors:** Maryam Delfan, Fatemeh Radkia, Raheleh Amadeh Juybari, Saeed Daneshyar, Mark ET Willems, Ayoub Saeidi, Anthony C Hackney, Ismail Laher, Hassane Zouhal

**Affiliations:** 1 Department of Exercise Physiology, Faculty of Sport Sciences, Alzahra University, Tehran, Iran; 2 Department of Physical Education, Hamedan University of Technology, Hamedan, Iran; 3 Institute of Applied Sciences, University of Chichester, Chichester, United Kingdom; 4 Department of Physical Education and Sport Sciences, Faculty of Humanities and Social Sciences, University of Kurdistan, Sanandaj, Kurdistan, Iran; 5 Department of Exercise and Sport Science, University of North Carolina, Chapel Hill, North Carolina, United States; 6 Department of Anesthesiology, Pharmacology, and Therapeutics, Faculty of Medicine, University of British Columbia, Vancouver, Canada; 7 Univ Rennes, M2S (Laboratoire Mouvement, Sport, Santé), Rennes, France; 8 Institut International des Sciences du Sport, Irodouer, France

**Keywords:** obesity, exercise training, Algomed, oxidative stress, adipo-myokine, Meteorin-like protein, insulin resistance

## Abstract

**Background:**

Dysregulation of adipocyte function occurs in obesity. Meteorin-like protein (Metrnl) is a newly discovered modulator of inflammation, metabolism, and differentiation of human adipocytes. The dietary supplement Chlorella Vulgaris (CV) reduces hyperlipidemia, hyperglycemia, and oxidative stress in clinical trials.

**Objectives:**

To explore the impact of 12 wks of interval resistance training (IRT) and CV supplementation on plasma levels of Metrnl and oxidative stress in males with obesity.

**Methods:**

Forty-four obese men (BMI: 32.0 ± 1.5 kg/m^2^, weight: 101.1 ± 2.2 kg, age: 23–35 years) were randomly assigned into 4 groups (*n* = 11/group): control (CON), CV supplement (CV), IRT, and CV + IRT (CVIRT). The IRT was performed for 12 wks (3 sessions per week). The treatment consisted of a daily intake of CV (1800 mg capsule) or placebo capsules. Blood samples were collected 48 hours before and after the interventions to analyze biomedical measurements.

**Results:**

The IRT and CVIRT groups had elevations in plasma Metrnl, superoxide dismutase, and total antioxidant capacity levels (all *P* < 0.0001), and reductions in malondialdehyde (*P* < 0.0001). Supplementation with CV significantly reduced malondialdehyde (*P* < 0.001) and increased total antioxidant capacity (*P* < 0.0001) but failed to alter superoxide dismutase or Metrnl (*P* > 0.05).

**Conclusions:**

Although IRT and its combination with CV hold promise for improving Metrnl levels and oxidative status in obesity, combining IRT and CV do not yield greater benefits than IRT alone. Although standalone CV supplementation could favorably impact certain markers of oxidative stress, the effectiveness of CV supplementation appears to have a relatively limited effect across assessed biomarkers and requires further investigation.

## Introduction

The global prevalence of obesity and its complications continues to increase and leads to greater rates of morbidity and mortality [1]. Abnormal or excessive fat accumulation and metabolic disturbances resulting from obesity are closely correlated with the burden of noncommunicable diseases such as type 2 diabetes, hypertension, dyslipidemia, atherosclerosis, cardiovascular diseases, and coronary artery disease [2].

Obesity is marked by chronic low-grade inflammation, which is associated with elevated levels of proinflammatory mediators that induce oxidative stress by promoting the overproduction of reactive oxygen species (ROS) and suppressing antioxidant defense mechanisms [3]. Increased levels of fatty acids lead to elevated oxidative stress, which in turn can result in dysregulation of adipose tissue [4] and cause detrimental endocrine and immune responses to further aggravate metabolic diseases [5]. Oxidative stress in fat depots is an early initiator of metabolic syndrome, highlighting the importance of regulating the redox state for managing obesity-related disorders [6].

The detrimental effects of inflammation-related oxidative stress can be mitigated by supporting antioxidant defenses, including glutathione peroxidase (GPx), catalase (CAT), glutathione reductase, reduced glutathione, and superoxide dismutase (SOD) [7]. Both enzymatic (e.g., GPx, CAT, SOD) and nonenzymatic (e.g., carotenoids, vitamins E and C, flavonoids) antioxidants neutralize oxidative reactions and protect cells from the destructive effects of ROS and delay the progression of chronic diseases [8].

Numerous studies have investigated the roles of the Meteorin-like protein (also known as Metrnl, Subfatin, Cometin, and Meteorin-β) as an adipo-myokine [9,10]. Metrnl improves lipid oxidation and glucose metabolism in skeletal muscle through the autocrine/paracrine signaling pathways [11] and protects against doxorubicin-induced oxidative stress and apoptosis through autocrine actions [12]. In addition, adipocyte-derived Metrnl counteracts obesity-related insulin resistance by improving adipose tissue function by stimulating metabolism and suppressing inflammation [13].

Metrnl expression is upregulated in response to physiologic stimuli, particularly by exercise in skeletal muscles and exposure to cold in white adipose tissue [14]. However, the association between obesity and circulating levels of Metrnl is unclear, as some studies reported increases in Metrnl levels in type 2 diabetes and obesity [[15], [16], [17]], whereas others reported decreases in Metrnl levels [[18], [19], [20], [21]].

The most widely recognized approaches for managing obesity are based on lifestyle modification programs (e.g., healthy eating habits, regular exercise, and behavioral interventions), pharmacological interventions, and surgical treatment [22]. Interest in dietary supplementation and adjunctive therapy has recently increased [23]. Natural marine sources, such as microalgae, are a promising source for the management of various diseases [24]. Microalgae are a source of macro- and micronutrients and are rich in a range of bioactive compounds, including lipids, proteins, carbohydrates, vitamins, carotenoids, dietary fiber, PUFAs, nucleic acids, essential amino acids, pigments, antioxidants, and other substances [23,25]. Chlorella Vulgaris (CV) is a unicellular freshwater microalga belonging to the Chlorellaceae family. It is used as a nutritional supplement with multifaceted health benefits [26] as shown by clinical studies indicating that supplementation with CV improves hyperlipidemia and blood glucose levels and protects against cancer and oxidative stress damage [26].

The traditional strategy for exercise in obese individuals focuses on endurance aerobic exercise training [27]. Recent research indicates that resistance training, in addition to developing muscle mass and strength, can enhance resting energy expenditure and fat metabolism, and optimize the weight loss process in obese people [28]. Physiologic adaptations to resistance training and alterations in body composition can be influenced by the number of sets, reps, intensity (percentage of one-repetition maximum [1RM]), volume, interset rest interval, and training frequency [29]. As an intermittent form of exercise, interval training, which comprises repeated bouts of effort interrupted with rest intervals or low-intensity activity for recovery, is also gaining popularity as participants find it more enjoyable than continuous exercise, making it a key strategy for long-term adherence to exercise programs [30,31].

Although CV supplementation and resistance training offer individual benefits [23,32], their combined effects on oxidative stress, antioxidant status, and adipo-myokine levels are not well understood. We investigated the effects of interval resistance training (IRT) and CV supplementation, alone and in combination, on plasma levels of SOD, malondialdehyde (MDA), total antioxidant capacity (TAC), and Metrnl in obese men.

## Methods

### Participants and research design

**FIGURE 1:**
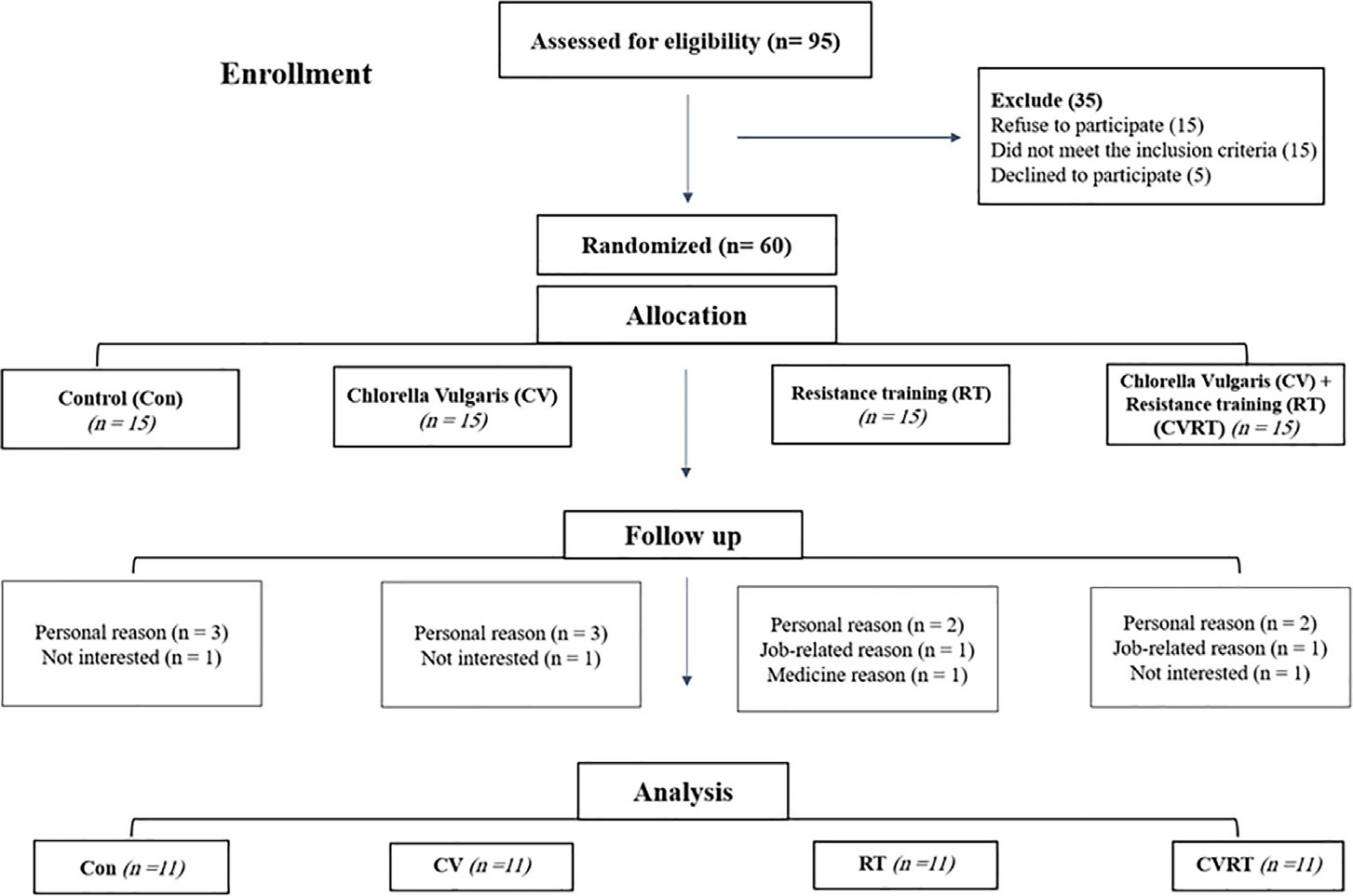
Flow diagram from enrolment to analysis of research participants. CON, control group with placebo; CV, Chlorella vulgaris group; CVIRT, Chlorella vulgaris plus interval resistance training group; IRT, interval resistance training group with placebo.

The study was performed as a double-blind randomized trial using a pretest and posttest design by enrolling obese men (*n* = 95, aged 23–35 y). The inclusion criteria were: a BMI of 30 kg/m^2^ or higher, lack of regular exercise participation, abstinence from smoking and alcohol consumption, and absence of pre-existing medical conditions such as hypertension, diabetes, cardiovascular disease, chronic kidney disease, or any other health issues. Based on these criteria, 60 participants were randomly allocated into 4 groups: control placebo (CON), CV supplement, IRT plus placebo group, and CV supplement plus IRT (CVIRT) group. The participants were provided with a detailed explanation of the protocols and guidelines, and written informed consent was obtained before their participation. After completing a medical history questionnaire, cardiologists and clinical exercise physiologists confirmed the eligibility of all participants. Participants who used drugs or other supplements did not follow daily supplement regimens, did not follow exercise training programs, or encountered new health concerns (*n* = 16) were excluded from the study. These exclusions resulted in a final enrolment of 44 participants (*n* = 11 for each group) (Figure 1).

### Ethical considerations

The trial followed the ethical guidelines of the Helsinki Declaration and was approved by the Ethics Committee of Sport Sciences Research Institute Tehran, Iran (IR.SSRI.REC.1400.1352). All participants were provided written informed permission following a thorough explanation of the study’s procedures and guidelines.

### Dietary adherence monitoring

Dietary adherence of participants was monitored during the 12-wk intervention study. The participants were provided with dietary recommendations and were required to adhere to their customary dietary patterns throughout the study.

### 1RM test

Participants in the IRT and CVIRT groups performed a 1RM test did not eat for 2 h before the test, abstained from alcohol for 48 h, and avoided caffeine for 12 h before the test. The 1RM was determined using the Brzycki Equation (1RM = weight lifted ÷ [1.0278 − (0.0278×repetitions to exhaustion)]) [33]. After a brief light-weight warm-up, participants were asked to select a weight that could be lifted for a maximum of 10 repetitions. The 1RM was calculated by incorporating the maximum weight lifted and the number of repetitions for each exercise [32].

### IRT program

Subjects in the IRT and CVIRT groups participated in a 12-wk IRT program that was monitored by exercise physiologists. The IRT protocol was implemented 3 times/week for 12 wks. Each session was 70 min, consisting of a 10-min warm-up, 50 min of core exercises, and 10 min of cool-down. The IRT protocol included 8 exercises, including seated leg extension, lying leg curl, leg press, back squats, chest press, barbell shoulder press, rowing, and front pulldown. The weight for lifting was 60% 1RM and was used for 3 sets that were separated by active rest intervals during which they did 15 repetitions at 20% of their 1RM [34].

### CV supplementation

Participants in the CV and CVIRT groups were given 6 capsules of CV (Algomed, Fardaye Sabz) containing two 300 mg capsules 3 times per day after meals so that the total consumption was 1800 mg/day [[35], [36], [37]]. Placebo capsules containing flour were given to the CON and IRT groups, at the same dosage as CV (two 300 mg capsules 3 times a day). Commitment to the supplementation schedule was tracked through regular check-ins during follow-up visits.

### Anthropometric assessment

Anthropometric characteristics were measured both prior to and 48 h after the 12-wk intervention. A digital scale with 0.1-kg precision was used to assess body weight without shoes and with little clothing (Seca), and a stadiometer with a 0.1-cm accuracy was used to measure body height (Seca). A bioelectrical impedance analyzer (Seca mBCA 555) was used to calculate the percentage of body fat. The BMI was calculated by dividing body weight by height squared (kg/m^2^).

### Blood sampling

**FIGURE 2:**
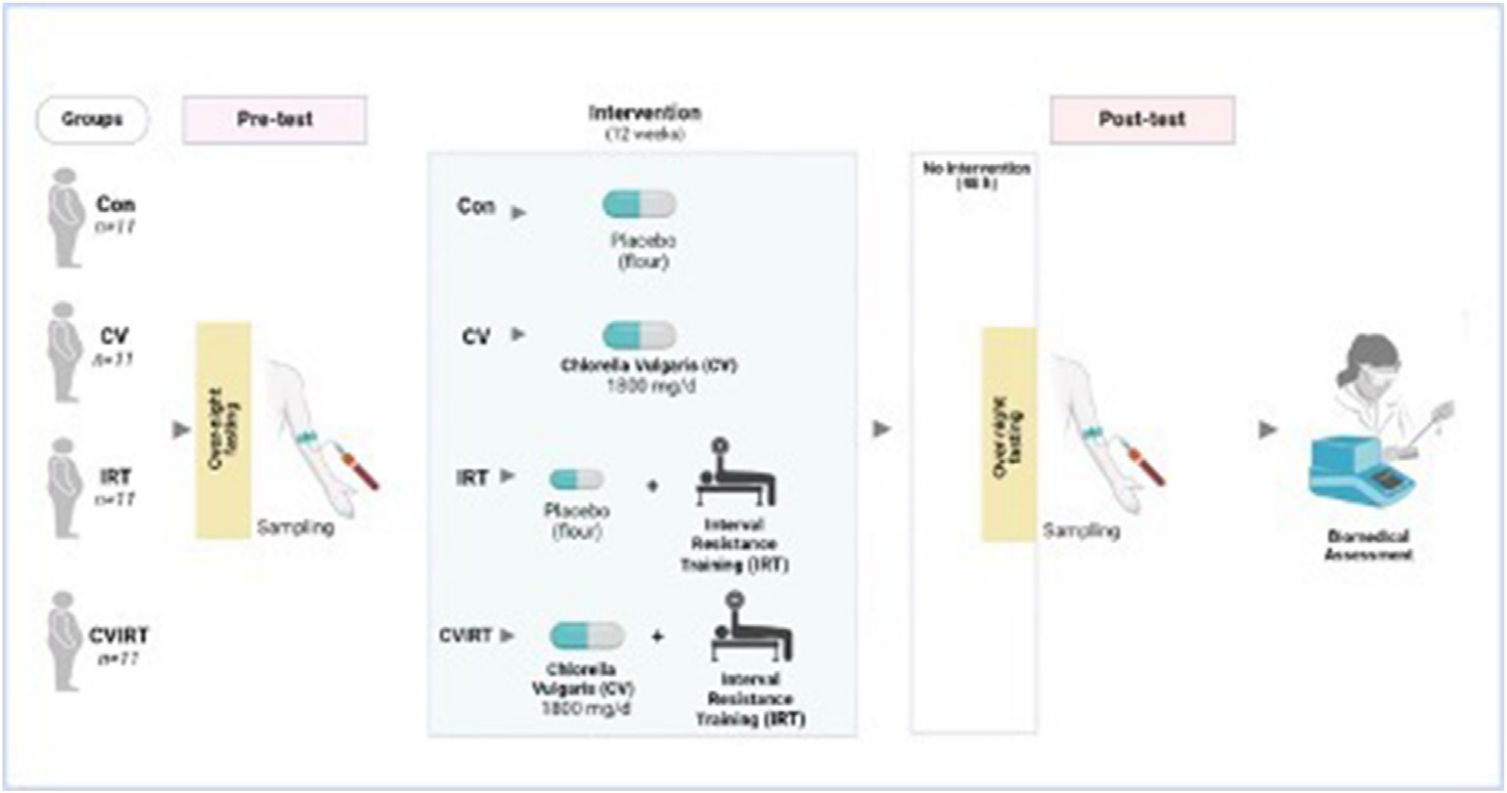
Schematic illustration of study methods. CON, the Control group with placebo; CV, Chlorella Vulgaris group; CVIRT, Chlorella Vulgaris plus interval resistance training group; IRT, interval resistance training group with placebo.

Blood samples were collected from the antecubital vein of participants 48 h before and after the intervention, while they had an overnight fast (Figure 2). The collection took place between 08:00 to 10:00. The samples were then transferred into tubes containing EDTA. After that, the plasma was separated by centrifugation at 1.03 g and 4°C for 10 minutes and stored at −20°C for later biomedical measurements.

### Biochemical parameter assessments

Plasma lipid profiles including triglyceride (TG), total cholesterol (TC), HDL, and LDL were assessed by a photometric method using commercial kits (Pars Azmun). Plasma glucose concentrations were evaluated by enzymatic colorimetric method using kits (Pars Azmun). Plasma insulin levels were measured by an ELISA method using an ELISA kit (Mercodia). The insulin resistance index was evaluated according to the HOMA-IR using the following formula: fasting plasma glucose (mmol/L) × fasting plasma insulin (μU/mL)/22.5.

Plasma levels of Metrnl were assessed with an ELISA kit (ZellBio GmbH). This assay had a sensitivity of 0.05 ng/mL with inter- and intra-assay variation of 16% and 8%, respectively. Plasma activities of the SOD were assessed using a Ransod kit (RANDOX) with a spectrophotometer at a wavelength of 505 nm. Plasma MDA levels used to measure lipid peroxidation were determined using a thiobarbituric acid reactive substances assay with a spectrophotometer at a wavelength of 532 nm. Plasma TAC levels were measured by the ferric-reducing antioxidant power assay based on the ability of pH (3.6) to reduce ferric (Fe^3+^) to ferrous (Fe^2+^) ions in the presence of 2,4,6-tripyridyl-s-triazine using a spectrophotometer at a wavelength of 593 nm.

### Statistical analysis

The normality of data was assessed using the Kolmogorov-Smirnov test, and the homogeneity of variances was determined with Levene’s test. The data were analyzed with repeated-measures two-way analysis of variance (ANOVA) followed by Tukey’s post hoc test. Partial Eta Squared (η^2^) was used to estimate effect size (ES). The partial eta squared for main effects was calculated from the ANOVA (η^2^p) and was interpreted as follows: 0.01 = small effect, 0.06 = medium effect, and 0.14 = large effect [38]. GraphPad Prism software (GraphPad Software) and SPSS 27 (SPSS Inc., Chicago) were used for statistical analysis. Data are reported as mean ± SD, and *P* < .05 was considered statistically significant.

## Results

Baseline values of study variables, including body weight, BMI, fat percentage, blood glucose, plasma insulin, HOMA-IR, HDL, LDL, TC, TG, Metrnl, SOD, MDA, and TAC levels were similar between the study groups (*P* > 0.05) (Table 1).

**TABLE 1:** Body composition and biochemical parameters of participants

		CON (*n* = 11)	CV (*n* = 11)	IRT (*n* = 11)	CVIRT (*n* = 11)
BM (kg)	Before	100.9 ± 2.6	102.3 ± 2.8	100.2 ± 1.6	101.3 ± 1.5
	After	102.6 ± 3.1	100.2 ± 3.6	97.2 ± 3.2∗∗∗	97.7 ± 3.4∗∗∗
	Δ	1.6 ± 4.5	−2.0 ± 4.7	−2.8 ± 4.1∗	−3.6 ± 4.0∗
	P^A^	0.51	0.39	0.091	0.044
BMI (kg/m^2^)	Before	32.3 ± 1.1	32.6 ± 2.0	31.2 ± 1.4	31.9 ± 1.5
	After	32.9 ± 1.9	32.0 ± 2.7	30.3 ± 1.0∗∗	30.8 ± 2.0∗
	Δ	0.64 ± 1.4	−0.66 ± 1.5	−0.90 ± 1.3∗	−1.1 ± 1.2∗
	P^A^	0.45	0.42	0.091	0.054
BF (%)	Before	34.0 ± 3.8	32.7 ± 3.3	35.2 ± 3.4	36.6 ± 2.2
	After	34.4 ± 3.3	30.8 ± 2.6	30.2 ± 3.0	31.0 ± 3.9
	Δ	0.44 ± 4.4	−1.88 ± 5.0	−5.01 ± 2.3∗	−5.62 ± 5.2∗
	P^A^	0.99	0.51	0.04	0.01
TG (mg/dL)	Before	256.3 ± 15.5	258.2 ± 16.7	256.8 ± 20.7	261.2 ± 19.1
	After	254.3 ± 12.8	255.1 ± 13.8	253.5 ± 14.1	245.6 ± 19.1
	Δ	−1.2 ± 4.3	−4.7 ± 4.2	−11.1 ± 4.5∗∗∗†	−12.5 ± 6.6∗∗∗††
	P^A^	0.48	0.016	0.0001	0.0001
TC (mg/dL)	Before	258.2 ± 16.7	250.9 ± 20.9	256.3 ± 13.6	252.9 ± 15.8
	After	257.0 ± 17.2	239.4 ± 18.5	239.4 ± 15.5	234.0 ± 11.1
	Δ	−1.2 ± 5.1	−11.4 ± 7.4∗	−16.8 ± 6.4∗∗	−18.9 ± 16.1∗∗∗
	*P*^*A*^	0.90	0.016	0.0001	0.0001
HDL (mg/dL)	Before	30.4 ± 6.4	31.5 ± 4.6	31.9 ± 10.6	28.8 ± 16.8
	After	31.0 ± 7.3	36.6 ± 5.6	39.2 ± 7.7∗	38.9 ± 6.9∗
	Δ	0.64 ± 4.9	5.09 ± 3.8	7.2 ± 4.0∗∗	−10.0 ± 4.5∗∗∗†
	P^A^	0.98	0.0016	0.0001	0.0001
LDL (mg/dL)	Before	172.0 ± 13.7	170.7 ± 13.9	172.3 ± 10.6	174.4 ± 16.8
	After	171.5 ± 13.5	163.9 ± 12.6	157.0 ± 8.3∗	155.5 ± 16.3∗
	Δ	−0.5 ± 5.5	−6.8 ± 3.8∗	−15.3 ± 6.7∗∗∗††	−18.8±4.5∗∗∗†††
	P^A^	0.99	0.001	0.0001	0.0001
BG (mg/dL)	Before	103.1 ± 13.1	105.7 ± 10.7	106.0 ± 5.7	108.3 ± 6.1
	After	97.4 ± 6.4	91.4 ± 4.5	87.8 ± 5.4∗∗	84.2 ± 6.7∗∗
	Δ	−5.7 ± 10.2	−14.2 ± 12.9	−18.1 ± 10.6 ∗∗	−24.1 ± 9.8∗∗∗
	P^A^	0.25	0.0001	0.0001	0.0001
Insulin (μU/L)	Before	19.4 ± 0.6	19.4 ± 0.7	19.4 ± 0.4	19.7 ± 0.5
	After	19.7 ± 0.5	18.4 ± 0.4∗∗∗	17.9 ± 0.5∗∗∗	17.4 ± 0.8∗∗∗††
	Δ	0.31 ± 1.0	−1.20 ± 0.1∗∗	−1.41 ± 0.5∗∗∗	−2.62 ± 0.7∗∗∗††
	P^A^	0.62	0.0001	0.0001	0.0001
HOMA-IR	Before	4.9±0.7	5.0 ± 0.5	5.0 ± 0.2	5.3 ± 0.4
	After	4.7 ± 0.3	4.1 ± 0.2∗∗	3.7 ± 0.2∗∗∗	3.7 ± 0.3∗∗∗†
	Δ	−0.21 ± 0.6	−0.90 ± 0.6∗	−1.3 ± 0.3∗∗∗	−1.6 ± 0.3∗∗∗†
	P^A^	0.0001	0.0001	0.0001	0.0001

Abbreviations: BF, Body fat; BG, Fasting Blood Glucose; BM, body mass; BMI, body mass index; CON, control group with placebo; CV, Chlorella Vulgaris group; CVIRT, Chlorella Vulgaris plus interval resistance training group; HDL, high-density lipoprotein; IRT, interval resistance training group with placebo, LDL, low density lipoprotein; TG, triglyceride; TC, total cholesterol; Δ: After – before trial; P^A^, *P* value based on intragroup comparison (After vs. before); *P*^Δ^, Adjusted *P* value based on intergroup comparison of Δ.Data are presented as the mean ± SD.

∗^,^∗∗^,^∗∗∗*P* < 0.5, *P* < 0.001, *P* < 0.0001 compared to the control group.

^†, ††, †††^*P* < 0.05, *P* < 0.001, *P* < 0.0001 compared to the Chlorella Vulgaris (CV) group.

### Body composition

Details of the body-composition data including body weight, BMI, and body fat percentage of the participants before and after the intervention of study groups are presented in Table 1. An intergroup analysis indicated that posttest body weights and BMI (but not body fat percent) were lower in IRT (5.2% for body weight, 7.9% for BMI) and CVIRT (4.8% for body weight, 6.3% for BMI) compared to the CON group (*P* < 0.05). The intragroup analysis showed that posttest values of body weight and fat percent in the CVIRT group were lower than pretest values (3.5% for body weight, 15% for fat percent; *P* < 0.05), also body fat percent in the IRT group were lower than pretest value (14%; *P* < 0.05). Further, differences in post–pre changes in body weight, BMI, and body fat percent in the IRT and CVIRT groups were lower than in the CON group (*P* < 0.05) (Table 1). The consumption of CV alone did not lead to a significant impact on body mass, BMI, or body fat percent (*P* > 0.05).

### Lipid profiles

Details of the lipid profile data including TG, TC, LDL, and HDL of each group at pretest and posttest are summarized in Table 1. A between-group analysis of TG showed that posttest TG levels of all groups were similar to the CON group (*P* > 0.05). However, within-group analysis indicated that posttest levels of TG were lower than pretest values (*P* < 0.05) in CV, IRT, and CVIRT groups. Changes in post–pre values of TG in IRT and CVIRT groups were lower than changes in the CON and CV groups (*P* < 0.05). Between-group analysis of TC indicates that posttest TC levels of all groups were similar to that of CON group (*P* > 0.05), and within-group analysis showed that posttest TC levels were lower than pretest values (*P* < 0.05) in the CV, IRT, and CVIRT groups. Changes in post–pre in TC in CV, IRT, and CVIRT groups were lower than in the CON group (*P* < 0.05). Between-group analysis showed that posttest LDL levels in the IRT and CVIRT groups were lower than in CON group (*P* < 0.05), whereas within-group analysis showed that posttest LDL levels were lower than pretest values (*P* < 0.05) in the CV, IRT, and CVIRT groups. Changes in post–pre values for LDL in the CV, IRT, and CVIRT groups were lower than in the CON group (*P* < .05), and the post–pre differences of LDL in the IRT and CVIRT groups were lower than in the CV group (*P* < 0.05). A between-group analysis showed that posttest HDL levels in the IRT and CVIRT groups were greater than that in CON group (*P* < 0.05), and within-group analysis showed that posttest HDL levels were higher than pretest values (*P* < 0.05) in CV, IRT, and CVIRT groups. Changes in post–pre values for HDL in IRT and CVIRT groups were higher than in CON group (*P* < 0.05), and post–pre data levels of HDL in the CVIRT group were higher than the CV group (*P* < 0.05) (Table 1).

### Glucose hemostasis

Pretest and posttest values for fasting glucose, insulin levels, and HOMA-IR levels are presented in Table 1. Between groups, analysis showed that posttest values of blood glucose, plasma insulin and HOMA were lower in the IRT and CVIRT groups than in CON group (*P* < 0.001), and that the posttest levels of insulin and HOMA-IR in the CVIRT group were lower than in the CV group (*P* < 0.05). The within-group analysis showed that the posttest levels of glucose, plasma insulin, and HOMA-IR were lower than the pretest levels in CV, IRT, and CVIRT groups. Differences in post–pre levels of blood glucose, plasma insulin, and HOMA in the IRT and CVIRT groups were lower than in CON group (*P* < 0.001) and the post–pre differences in plasma insulin and HOMA (but not blood glucose) in the CVIRT group were lower than in the CV group (*P* < 0.05) (Table 1).

### Plasma levels of Metrnl

**FIGURE 3:**
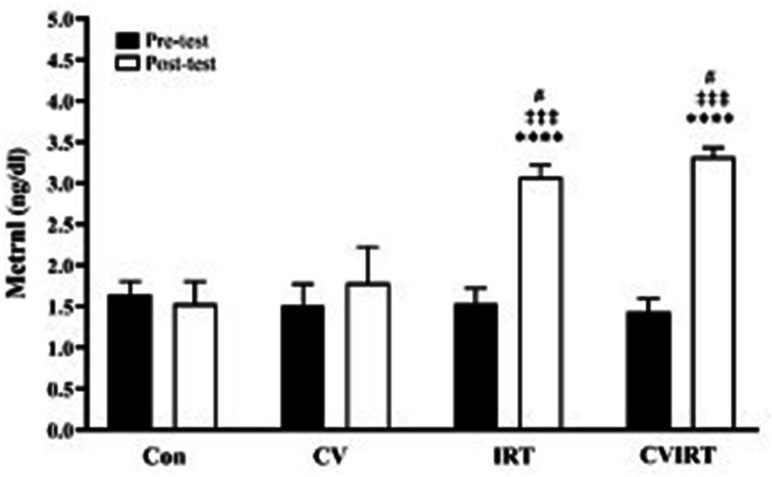
Plasma levels of Metrnl. Data are presented as the mean ± SD. CON, the Control group with placebo, CV, Chlorella Vulgaris group; CVIRT, Chlorella Vulgaris plus interval resistance training group; IRT, interval resistance training with placebo. ∗∗∗∗ indicate *P* < 0.00001 from pretest; ‡‡‡ indicate *P* < 0.0001 from posttest of Con group; # indicate *P* < 0.05 from posttest of CV group.

The two-way repeated measures ANOVA indicated an interaction between group × time (η^2^ = 0.87, *P* < 0.0001). Additionally, there were significant main effects of time (η^2^ = 0.83, *P* < 0.0001) and group (η^2^ = 0.85, *P* < 0.0001). An intragroup comparison demonstrated that both IRT alone and IRT plus CV increased the plasma levels of Metrnl in obese men (*P* < 0.0001). However, CV alone did not change plasma levels of Metrnl (*P* = 0.091). Accordingly, an intergroup comparison indicated that both the IRT and CVIRT groups had increased levels of Metrnl compared to CON (*P* < 0.0001) and CV groups (*P* < 0.05). However, there was no difference in Metrnl levels between the CVIRT and the IRT groups (*P* > 0.05) (Figure 3).

### Antioxidant status

#### Malondialdehyde

**FIGURE 4:**
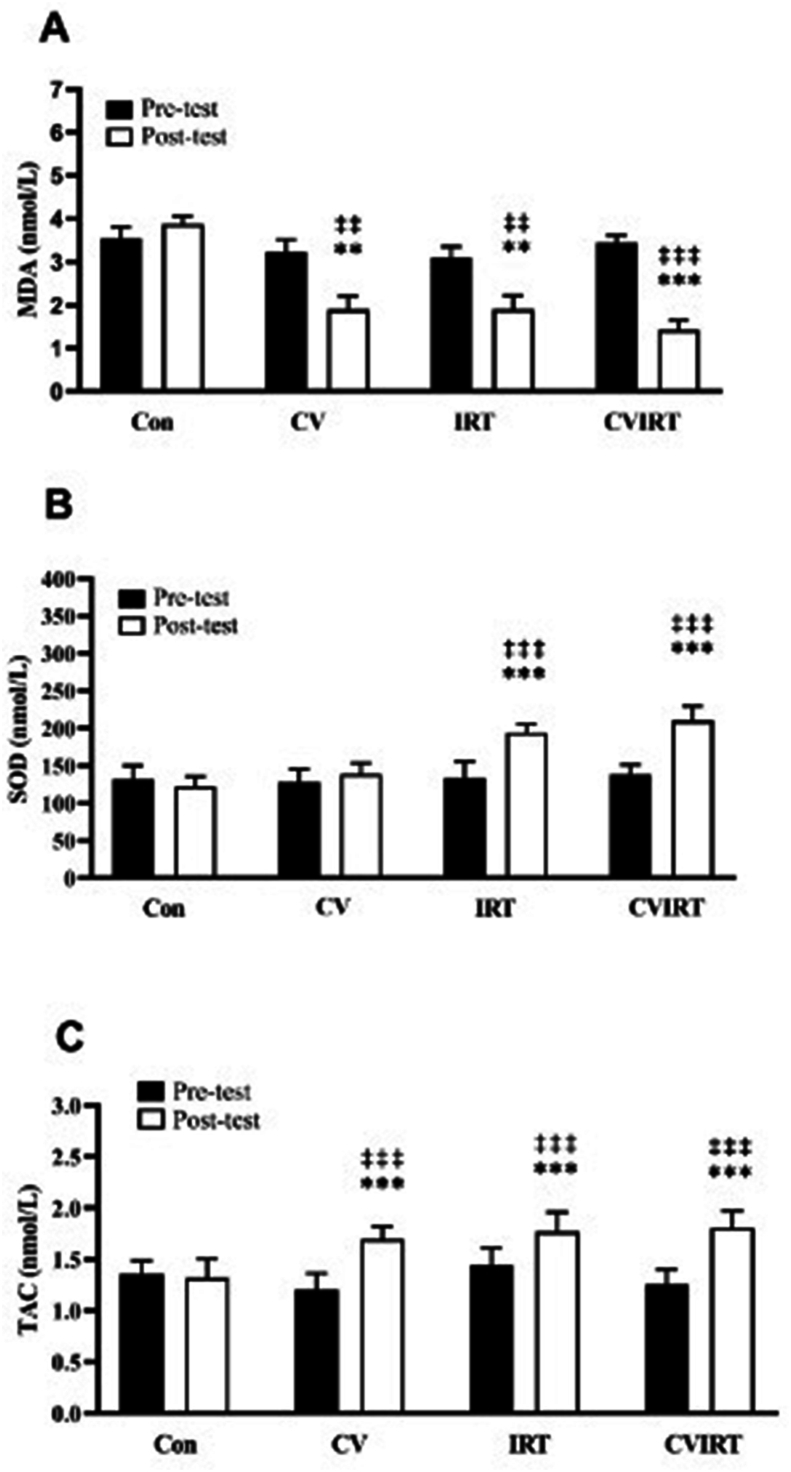
Serum concentrations of Malondialdehyde (A), Superoxide dismutase (B) and total antioxidant capacity (C). Data are presented as the mean ± SD. CON, control group with placebo, CV, Chlorella Vulgaris group; CVIRT, Chlorella Vulgaris plus interval resistance training group; IRT, interval resistance training with placebo. ∗∗,∗∗∗ indicate *P* < 0.001 and *P* < 0.0001 from pretest; ‡‡ , ‡‡‡ indicate *P* < 0.001 and *P* < 0.0001 from posttest of CON group.

Two-way repeated measures ANOVA indicated a group × time interaction (η^2^ = 0.33, *P* = 0.0004). There were significant main effects of time (η^2^ = 0.44, *P* < 0.0001) and group (F (η^2^ = 0.46, *P* < 0.0001). The intragroup comparison indicated that IRT, CV, and IRT + CV have all led to a significant reduction in MDA levels (*P* < 0.01). The intergroup comparison showed that the CV, IRT, and CVIRT groups all showed a significant decrease in MDA levels compared to the CON group (*P* < 0.001) (Figure 4A).

#### SOD

The two-way repeated measures ANOVA showed that there were significant group × time interactions for SOD levels (η^2^ = 0.67, *P* < 0.0001), with significant effects of time (η^2^ = 0.66, *P* < 0.0001) and group (η^2^ = 0.78, *P* < 0.0001). An intragroup comparison showed that both IRT alone and IRT + CV increased SOD levels (*P* < 0.0001). However, CV alone did not affect SOD plasma levels in obese men (*P* > 0.05). A comparison between the groups, both the IRT and the CVIRT groups showed a significant increase in SOD levels compared to the CON and the CV groups (*P* < 0.0001). No significant difference was observed between the CVIRT group and the IRT groups (*P* = 0.225) (Figure 4B).

#### TAC

The two-way repeated measures ANOVA indicated a significant group × time interaction (η^2^ = 0.55, *P* < 0.0001), with significant main effects of time (η^2^ = 0.73, *P* < 0.0001) and group (η^2^ = 0.43, *P* < 0.0001). An intragroup comparison showed increased plasma TAC levels in IRT, CV, and CVIRT groups (*P* < 0.0001). When comparing the groups, the CV, IRT, and CVIRT groups exhibited increases in TAC compared to CON group (*P* < 0.01), with no differences between the CV, IRT, and CVIRT groups (*P* = 0.95) (Figure 4C).

## Discussion

This study examined the effects of IRT and CV supplementation, both independently and in combination, on Metrnl, MDA, SOD, and TAC in obese men. The main findings of this study are that 1) IRT alone or in combination with the CV increased plasma levels of TAC, SOD, and Metrnl and reduced plasma MDA levels, and *2*) CV alone reduced plasma levels of MDA and increased plasma TAC levels, without affecting levels of SOD and Metrnl. The combination of IRT and CV supplementation does not provide greater benefits compared to the execution of IRT alone.

Our study demonstrates that IRT, either alone or in combination with CV, reduced body weight and BMI. However, CV supplementation alone did not affect body weight or BMI. Furthermore, CV, IRT, and their combination improved the lipid profile by a reduction in LDL, TC, and TG levels, and also increased HDL levels. Additionally, insulin resistance index (HOMA-IR) was improved by the effects of IRT, CV, and the combination of IRT and CV.

Metrnl is a hormone (secretory protein) that can be selectively activated in tissues by specific physiologic stimuli [14]. For example, thermogenic triggers, particularly acute and chronic exposure to cold, upregulate Metrnl expression in adipose tissues, whereas muscle contraction promotes Metrnl production in skeletal muscle [9]. Metrnl enhances the browning process of white adipose tissue, thermogenesis, and energy expenditure, and also improves glucose intolerance [39]. Levels of Metrnl proteins are increased in individuals diagnosed with type 2 diabetes and obesity [[15], [16], [17]], with a positive correlation between serum concentrations of Metrnl and metabolic indicators such as BMI, waist circumference, fasting blood glucose, Hemoglobin A1c (HbA1c), and HOMA-IR [17]. On the contrary, other studies have reported lower levels of Metrnl in individuals with prediabetes, diabetes, or obesity [[18], [19], [20], [21]] and suggested that Metrnl levels negatively correlate with fasting blood glucose, fasting insulin, HOMA-IR, and HbA1c [19,20].

Plasma levels of Metrnl increased in response to both IRT and IRT plus CV in our study. Electrical stimulation-induced resistance exercise in rats increases serum Metrnl [40], whereas 8 wks of circuit resistance training increases plasma Metrnl levels in patients with type 2 diabetes mellitus [41]. However, a study by Saeidi et al. [42] reported that 12 wks of resistance training failed to increase plasma Metrnl levels in obese men. The possible reason for this difference in results between our study and that of the Saeidi et al. [42] study may be related to differences in subject characteristics: the participants of our study were younger and had lower BMI, and also Saeidi et al. [42] study used traditional resistance training whereas our study used an interval protocol of resistance training. It is possible that IRT has more effect on Metrnl production compared to traditional resistance training. This possibility is supported by a previous study showing that IRT had a superior effect on adipokine than the traditional one [43].

Metrnl has a protective role in inflammation, insulin resistance, and lipid metabolism [39]. Improvements in lipid profiles and insulin resistance in the RT and CVIRT groups in our study were related to increases in plasma Metrnl levels, suggesting a negative relation between Metrnl and these variables, indicating that increases in Metrnl induced by resistance training could mediate the benefits of resistance training against obesity-related complications such as insulin resistance. The source of Metrnl released into circulation by resistance training is unclear, with some studies indicating that exercise increases Metrnl mRNA expression levels in skeletal muscle [[44], [45], [46]] and also in the gastrocnemius muscles of rats following 4 wk of resistance training [40].

Several studies have highlighted the role of oxidative stress at the onset and progression of obesity-related inflammation [47]. Protection against the harms of oxidative stress is provided by antioxidant defenses, including GPX, SOD, and CAT [48]. A surge in lipid peroxidation is a hallmark of oxidative stress [49], as monitored by increases in MDA levels [6]. Our findings show that IRT and CV, both independently and in combination, reduced MDA levels. Administering CV reduced DNA damage and MDA levels in diabetic rats [50].

Our study also demonstrates that IRT and CV, whether undertaken separately or in combination, increase TAC levels in obese men. The antioxidant activity of CV and its ability to regulate antioxidant status has been reported in several studies [12,51,52]. CV decreases lipid membrane peroxidation by suppressing the production of ROS, primarily by scavenging free radicals or by augmenting cellular antioxidant defenses [12,51]. The anti-inflammatory effect of CV is due to having polyphenolic compounds such as carotenoids, polysaccharides, chlorophyll, and polyphenols [26]. The decreases in MDA levels in our study are likely mediated, at least in part, by the polyphenols contained in CV.

Our study did not show increases in SOD levels in obese men treated with CV, but SOD levels were increased by resistance training alone or in combination with CV. Trace minerals such as zinc, copper, selenium, iron, and manganese are cofactors in the functioning of antioxidant enzymes such as GPX, SOD, and CAT [53]. The presence of these components in CV can promote health by modulating the signaling pathway to combat oxidative stress [12]. The lack of effect of CV on SOD levels could be due to the insufficient dose/treatment time used.

Our findings are supported by several studies reporting that exercise increases SOD and reduces MDA levels. Resistance training for 12 wk total increased antioxidant capacity, which persisted even after 3 mon of detraining, in older women with an average BMI of 28.3 kg/m^2^ [54]. Furthermore, 6 months of resistance exercise lowered exercise-induced oxidative stress, regardless of adiposity, in overweight older individuals [55].

Supplement prescriptions for health promotion entail the consideration of various factors such as dosage, individual differences, health status, treatment plans, and the quality and absorption of supplements, all of which impact results [56,57]. Although antioxidant supplementation can regulate exercise-induced oxidative stress, administering antioxidants may have negative effects on individuals with an already optimal redox state. Furthermore, prolonged excessive antioxidant intake can interfere with physiologic adaptation to exercise by suppressing redox-sensitive signaling pathways and mitochondrial biogenesis [58]. Additionally, overloading the cell with high doses of antioxidants diminishes the beneficial effects of exercise training and interferes with crucial ROS-mediated physiologic processes [59]. Research on CV as a plant-derived supplement has investigated dosages ranging from 500 mg to 8 g per day across different purposes and demographic groups, highlighting the necessity for personalized, condition-specific trials to identify the most effective doses [35,60]. We found no adverse effects of CV supplementation on exercise benefits in our study group, although combining CV with exercise did not significantly improve parameters when compared with exercise alone. Their combination showed a positive trend without statistical significance, suggesting adjustments in duration or dosage might alter results, which requires further investigation.

### Study limitations

There are several limitations in our study: *1*) the exclusive focus on relatively young obese men limits applicability to other demographic groups, warranting caution in generalizing the results; *2*) the outcomes most certainly could be affected by individuals' dietary intake and activity levels, which were not controlled in the study; *3*) although bioelectrical impedance analyzers provide a valuable and noninvasive method for estimating body composition, they have limitations in terms of accuracy and applicability to individual characteristics [61]. Therefore, it is best to use them in conjunction with other more precise analysis methods’ and, *4*) it is recognized that the within-group sample size was small thereby limiting the statistical power of our outcomes.

In conclusion, this study demonstrates the promising effects of IRT in combination with CV supplementation on ameliorating oxidative stress and enhancing beneficial adipo-myokine levels in young adult obese men. Although the combined approach showed favorable results, it did not demonstrate superior effects compared to IRT alone. Furthermore, although standalone CV supplementation may lead to an improvement in some oxidative stress markers, further research is necessary to fully evaluate the efficacy of CV supplementation or its synergistic effect with exercise training.

## Author contributions

The authors’ responsibilities were as follows – MD, AS, HZ: designed the study; MD, FR, AS: conducted the study; SD: analyzed the obtained data; MD, SD, RAJ: wrote the first draft of the manuscript; and all author: read and approved the final manuscript.

## Conflict of interest

The authors report no conflicts of interest.

## Data Availability

The datasets generated for this study are available on request to the corresponding authors.
